# Damage and Failure of Axonal Microtubule under Extreme High Strain Rate: An In-Silico Molecular Dynamics Study

**DOI:** 10.1038/s41598-018-29804-w

**Published:** 2018-08-16

**Authors:** Yuan-Ting Wu, Ashfaq Adnan

**Affiliations:** Department of Mechanical and Aerospace Engineering, The University of Texas at Arlington, Arlington, TX 76019 USA

## Abstract

As a major cytoskeleton element of the axon, the breaking of microtubules (MTs) has been considered as a major cause of the axon degeneration. High strain rate loading is considered as one of the key factors in microtubule breaking. Due to the small size of microtubule, the real-time behavior of microtubule breaking is hard to capture. This study employs fully-atomistic molecular dynamics (MD) simulation to determine the failure modes of microtubule under different loadings conditions such as, unidirectional stretching, bending and hydrostatic expansion. For each loading conditions, MT is subjected to extreme high strain rate (10^8^–10^9^ s^−1^) loading. We argue that such level of high strain rate may be realized during cavitation bubble implosion. For each loading type, we have determined the critical energy for MT rupture. The associated rupture mechanisms are also discussed. We observed that the stretching has the lowest energy barrier to break the MT at the nanosecond time scale. Moreover, the breakage between the dimers starts at ~16% of total strain when stretched, which is much smaller compared to the reported strain-at-failure (50%) for lower strain rate loading. It suggests that MT fails at a significantly smaller strain states when loaded at higher strain rates.

## Introduction

**Figure 1:**
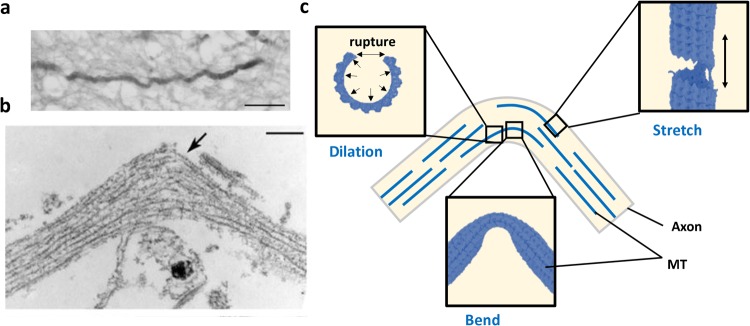
(**a**) Undulated human axon under traumatic brain injury (post-mortem)^3^. Scale bar: 25 μm. (**b**) TEM image of axon after stretching experiment^3^. Arrow shows curling configuration of MT in the original paper. Scale bar: 500 nm. (**c**) Schematics of the possible mechanical failure modes of MT due to bending of an axon. The three subfigures are added to identify possible deformation and failure process of MT.

Microtubules (MTs) are one of the major structural components of the cytoskeletons of axon. Axons are responsible for long-range neuronal communication. MTs in an axon help to transport molecules^1,2^ from end to end. Thus, the breaking of microtubules can lead to the failure of local equilibrium, which may later cause swelling or rupture of the axon^3^ and lead to disruptive transmission of action potential^4,5^. Failure of MT also leads to the disconnection and retraction of axon (Fig. 1). Experimentally, it is found that various type of loading conditions such as extension^6^, bending^7–9^ or combined loading may cause rupture of MTs. For mechanically loaded MT in an axon, research indicates that the loading rate (strain rate) is critical to determine whether it leads to complete axonal failure or local axonal damage^9–11^.

While axonal damage can occur by various loading types, recently, cavitation implosion is being investigated as possible mechanisms for axonal damage^10–14^. The characteristic time scale to collapse a millimeter bubble is below millisecond. The associated loading rate can be estimated by the given cavitation collapsing speed. Considering most bio plasma is nearly incompressible under the given pressure change, the velocity of the surrounding can be estimated with the assumption of incompressibility and spherical symmetry as

1 $$u(r)=\dot{a}{a}^{2}/{r}^{2},$$

where *u*, *a*, $\dot{a}$, and *r* are radial fluid velocity, bubble radii, bubble surface velocity, and the distance from bubble center. Thus, the local strain rate on the radial direction, $\dot{\varepsilon }$, can be computed by the differentiating *u*(*r*) with respect to *r* as

2 $$\dot{\varepsilon }=\frac{du(r)}{dr}=-\,2\dot{a}{a}^{2}/{r}^{3},$$

Equation 2 suggests that the largest strain rate will be felt at the bubble surface with a magnitude equals to $-2\dot{a}/a$. If a 1 mm bubble collapses within 100 μs, then $\dot{a}$ is roughly 10 m/s. Under such scenario, the strain rate exerted by the surrounding fluid will be ~10^6^ s^−1^ at 10 μm range and ~10^8^ s^−1^ at 100 nm range. This estimation agrees with the studies focused on possible cavitation damage^15^. From the above equations, we can have a rough idea of how robust the cavitation can be. Thus, many researchers are now investigating the full mechanism of it during events like traumatic brain injury (TBI)^15–19^. In our previous study^17^, we reported that the cavitation bubble collapse can be strong enough to rupture sub-cellular structure, which has led us here to investigate the vulnerability of microtubule.

One should notice that MTs are capable of rapidly reconfiguring themselves in response to the evolving cellular demands such as chromosome segregation, organelle transport and scaffold formation for transport and morphological changes. They do it through a self-assembly mechanisms, where the terminal molecules of the microtubule randomly switches between alternate cycles of slow growth and rapid shortening such that individual αβ-tubulin heterodimeric subunits undergo net addition and loss, respectively^6^. The overall stability of MTs is controlled by this reconfiguration process that takes place over the characteristic time on the order of seconds^20^. Thus, a loading process that lasts several seconds may strongly interact with the thermodynamic assembly-disassembly process of MT. The corresponding deformation and failure mechanisms of MT will also be guided by the coupled effect. We argue that the loading type that we have focused here occurs significantly faster than the thermodynamic assembly-disassembly process of MT. As such, these two events do not interact with each other.

Due to the difficulty to observe MT’s deformation and failure in real time with necessary structural detail, efforts are made to develop computational models spanning between sub-atomic scale and macroscopic continuum scale. Although a sub-atomistic scale fist-principal quantum mechanics (QM) model of MT may provide finest structural detail, obtaining even one periodic section of MT level data out of such simulation method is daunting due to the high computational cost. Each periodic section of MT (13 alpha-beta tubulin dimer) consists of around 170,000 atoms, which is way beyond the current capability of QM simulation. Currently, fully-atomistic molecular dynamics (MD) model is the most feasible simulation tool that can generate meaningful data at the atomistic resolution^21–26^. Recently, it has been demonstrated that a fully-atomistic model containing the entire *αβ*-dimer ring structure of MT (13 alpha-beta tubulin dimer) is computationally demanding but feasible^24^. By using the periodic boundary conditions, one can perform MD simulation of a virtually infinitely long MT. Using currently available high performance computational power, it is now possible to include more than one periodic sections of MT. However, due to the overwhelming detail, at the temporal scale, fully atomistic MD is still not capable of simulating more than few nanoseconds. To capture dynamics of MT for a longer time, coarse grain MD (CGMD) are often used. The CGMD method allows us to run a simulation with larger domain size and longer duration with less atomistic detail^26–32^. Depending on the level of coarse graining, CGMD can caputre the behavior of MT from microseconds to milliseconds. The continuum level models can describe the behavior of MT even at the level of seconds or beyond but such method is unable to capture atomistic detail^23,33–37^. It is evident that atomistic level simulations can capture events at the atomistic resolution but only for nanosecond-range duration. Continuum level simulations, on the other hand has no limitation on the experimentally relevant time scale but lack configurational detail. As such, the characteristic length time and length scale of the problem drive the choice of modeling platform.

**Figure 2:**
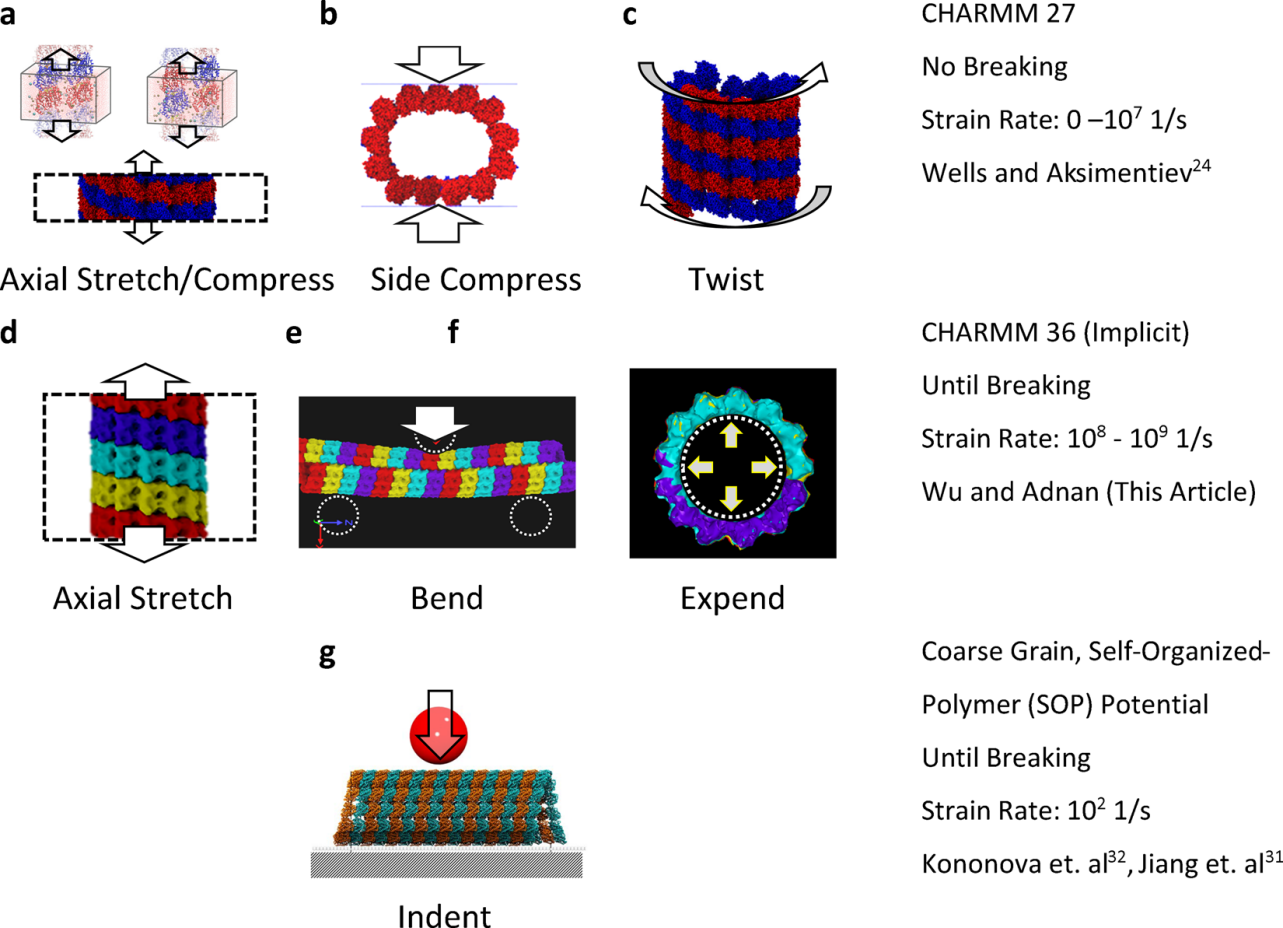
Comparison between the current study with the existing studies to examine the mechanical characteristic of microtubule using molecular simulations.

Our current effort is driven by our recent findings on the collapse of cavitation bubble in the perineuronal net (PNN) of brain, the region of Brain’s extracellular matrix located adjacent to neurons^17,38^. What we found in that study is that when cavitation bubble collapses, a high velocity localized flow is developed near the collapse side. Hypothetically, if such a jet is impinged-on the surface of a neuron cell, a high strain-rate jolt will be felt by neuron and its cytoskeleton elements such as MT. Our hypothesis is that such a loading can lead MT to experience axial and bending loads. Since stretch injury triggers microtubule instability^6^ and microtubule instability is often associated with protofilament segregation, it is also relevant to study what level of mechanical force is needed to cause protofilament separation. For this, MT needs to be subjected with hydrostatic expansion. The length scale (~100 nm) and time scale (~ns) involved during the final stage of cavitation collapse suggest that full-atomistic MD should be right simulation platform. As such, in this study, we simulated stretching, bending, and expansion simulations in full-atomistic MD. We tested different strain rates for the stretching test. We documented the breaking patterns of the simulated MT in all modes along with the potential energy profile and force profile. Compared to earlies studies where only a few dimers were used to construct MT, our study revealed unique failure mechanisms of MT in response to mechanical loading rate. The comparison of this study with most relevant previous studies is shown in Fig. 2.

## Results and Discussions

### Stretching Test

For the stretching test, we tested three different strain rates, 2.5 × 10^8^ s^−1^, 1 × 10^9^ s^−1^ and 4 × 10^9^ s^−1^, based on the estimated cavitation collapsing strain rate. Four *αβ*-dimer rings of MT were included. After equilibration (see the Method section for more detail), a velocity field was applied along the z direction corresponding to the desired strain rate. The simulated time were set at 0.25 ns, 0.75 ns, and 3 ns, respectively, to achieve 75% strain with the assigned strain rate.

**Figure 3:**
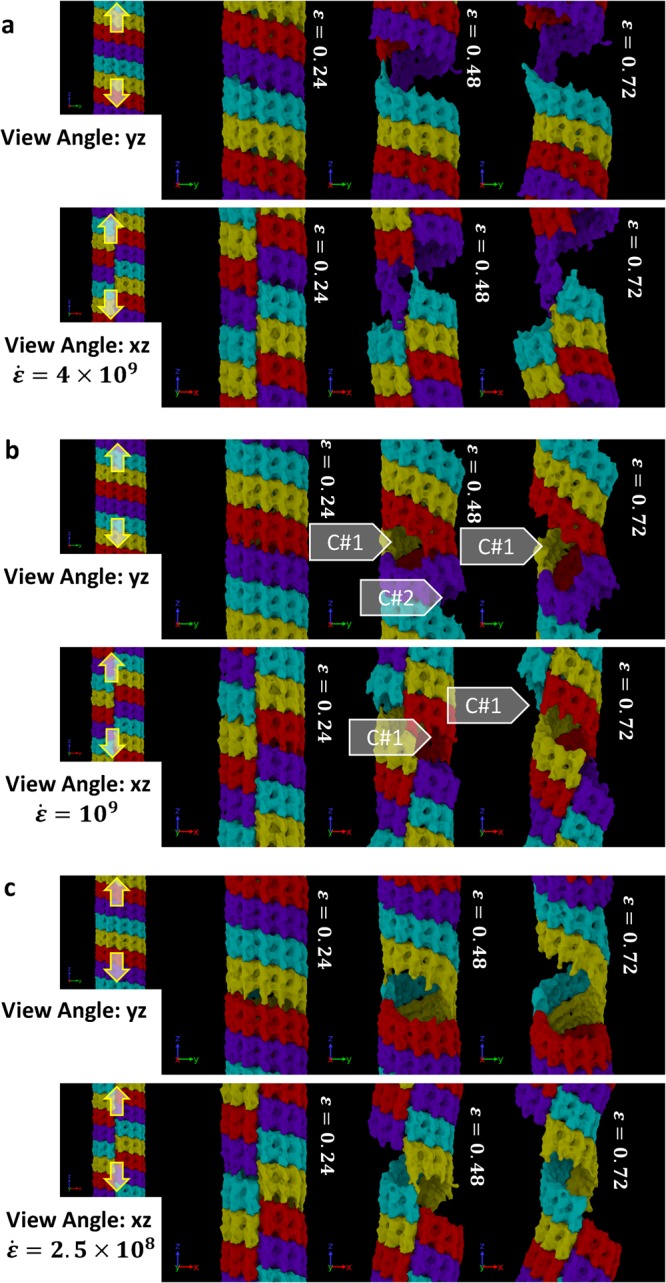
Breaking pattern of MT molecules during axial stretch at different strain rates equal to (**a**) 4 × 10^9^ 1/s, (**b**) 1 × 10^9^ 1/s, and (**c**) 0.025 × 10^9^ 1/s. The filled arrow-heads point to the possible failure nucleation sites. The figures are showing more than one periodic images for visualizing the cracking site that cross the periodic boundary. Each periodic image contains 4 tubulin rings (marked in 4 different color). Note the rupture mechanisms of MT beyond *ε* = 0.24. Failure via inter-dimer (α–β) separation along the longitudinal direction is clearly visible.

The results are reported in Fig. 3. In Fig. 3, different *αβ*-dimer rings are marked in different colors for easily pinpointing the failure location. Note that each ring contains 13 *αβ* tubulin dimers. The results suggest that the connection between the α and β tubulins are stronger than the connection between the dimers since the breakage happened in between *αβ* dimer in all the stretch case simulated. In all three cases, an interfacial crack can be observed at around 20% strain. The crack then propagates along the interface separating dimer rings and finally unwinds the helical structure. In the fastest strain rate case (i.e 4 × 10^9^ s^−1^), the unwinding continues with the increasing strain state. For the case of 10^9^ s^−1^, more than one cracks were simultaneously formed (marked in Fig. 3b, crack #1 and #2 but every crack did not fully propagate around the circumference of MT. The larger crack eventually traveled across the junction intersecting the protofilament-protofilament interface (along z direction) and helical dimer ring interface. Due to the helical structure, the tangential connection at the junction is not α-to-α and β-to-β; it is α-to-β and β-to-α. Interestingly, the tangential crack shortly reroutes to the axial direction (marked in Fig. 3b, crack #1). For the 2.5 × 10^8^ s^−1^ case, only one crack was formed and propagated along the helical line between the dimer rings.

**Figure 4:**
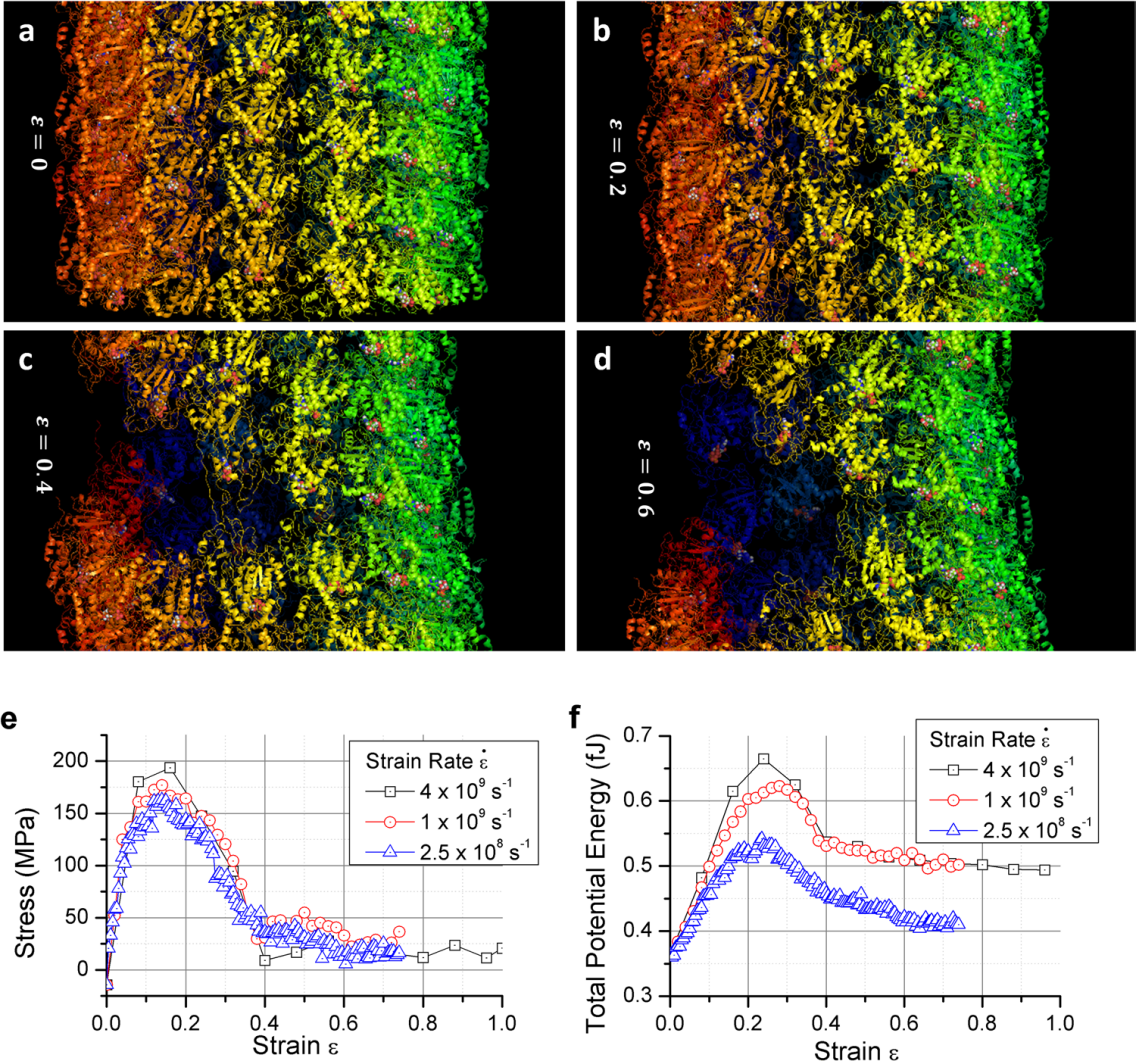
(**a**–**d**) Ribbon diagrams of MT breakage under a 10^9^ s^−1^ strain rate. Organic molecules that are not protein are plotted by atoms. Different colors are used across the ring to ensure the visibility (**e**) Stress profile of the MT under three different strain rates. The stress is calculated by the total virial stress with a constant approximated 325 nm^2^ area. (**f**) The total potential energy of the simulated system (4 tubulin ring) under three different strain rates. For both figure, data points are logged with a 20 ps interval.

To visualize the deformation and failure mechanisms of MT near the failure sites, magnified view of MT represented with “ribbon” form is shown in Fig. 4a–d. Figure 4a–d highlights the major breakage of the case of 10^9^ s^−1^ (marked as Crack #1 in Fig. 3b). One can observe that the protein structures (α-helical or β-sheet) near the breaking site are elongated during the axial (stretch) load. It can be noted that a different color scheme is used to represent the atoms/dimers here for clarity.

We calculated the virial stress (*zz*) and the total potential energy at each applied strain state and the results are plotted in Fig. 4e,f. Note that the total virial stress is calculated by summing the atomic-level virial (*zz*) over the entire simulation box and then by dividing the sum with the volume of MT. The volume of MT is approximated by multiplying the total length of the simulation domain with the estimated cross sectional area of MT (325 nm^2^). In principle, MT’s cross section is not fixed along its length. As such, the reported failure strength of MT should be regarded as an estimate. What is more meaningful from this study is the deformation mechanisms in MT that leads to failure. As shown in Fig. 4, MTs exhibit elastic deformation roughly up to 5% strain. It then starts to yield and eventually breaks at 16–20% strain. Such trend is consistently observed in all three cases studied here. By using the data during elastic deformation, the Young’s modulus (*zz*) and viscosity (*zz*) of MT are computed. It is found that the Young’s modulus of MT is 2.3 GPa and the viscosity is roughly 16 mPa-s. As one can observe in Fig. 4f, the total potential of the 2.5 × 10^8^ s^−1^ is growing slower after the breaking point comparing to the other two higher strain rate cases. We infer that such trend is associated with number of crack formation and propagation during tensile loading.

### Bending Test

**Figure 5:**
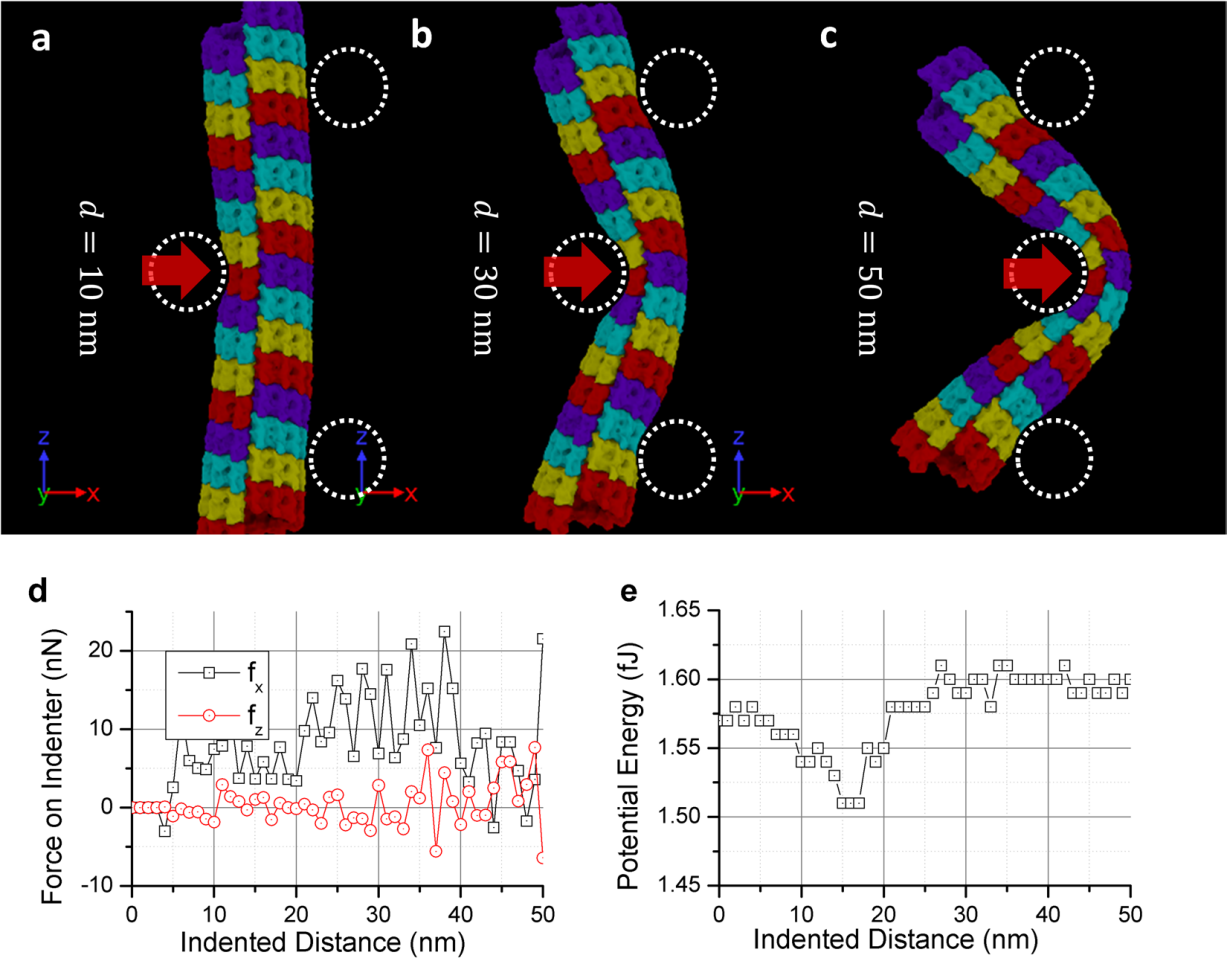
(**a**–**c**) The deformed MT after the three-points bending test. The dotted circles marked the artificial indenter and support we inserted. There are 16 tubulin rings used in the test. (The colors are used repeatedly). (**d**) Forces are recorded on the center indenter during the bending test (sign reversed). (**e**) The total potential energy of the simulated system under the bending test. For both figures, data points are logged with a 20 ps interval.

To obtain the bending response of MT, we included 16 *αβ*-dimer rings of MT in our model and numerically performed a typical 3-points bending test. The two support cylinders are placed 90 nm apart, and the indenter cylinder is placed in the middle. The support and indenter cylinders interact with the MT atoms through a Lennard-Jones (LJ) potential. (see the Method section for more detail of the definition of the cylinders) All cylinders are10 nm in radius and placed at least 2 nm away from the MT (marked as dotted line in Fig. 5a–c). Since computational cost for simulating 16 *αβ*-dimer rings is significantly higher, we only performed one simulation for the bending test. The indenter cylinder continues to push the MT until a total distance of 50 nm with the indenting speed of 50 m/s. The total simulated time is 1 ns.

The MD snapshots during deformation and the associated force-displacement plots are shown in Fig. 5a–c. It can be observed that Similar to Fig. 3, the color scheme used here is to distinctly show the *αβ*-dimer ring structures. Note that bending simulations are done without any periodic boundary conditions. In our study, the indenting direction is set at 90 degrees against the dimer ring junction. By doing this, we have ensured that the bending load resulted in maximum local shell-bending at the junction. At the end of the simulation, the MT bent more than 90 degrees; however, no sign of breakage between the dimers were observed. The MT cylinder shell buckled, and the interior walls eventually touched the other side as bending continued.

We documented the force on the indenter and the systematic potential energy in Fig. 5d,e. It can be observed that the force on the *z* directing (the original MT axial direction) only oscillates near zero while the force in the *x* direction (the indenting direction) keeps increasing when the indenter goes deeper. The bent shape agrees with the illustration provided in the literature^30,33^. However, comparing it to other literatures that used a spherical indenter with loading rate of 1 μm/s speed, we found that the force on our indenter is roughly 10 times larger than the reported values in the literatures^31,32^. Also, it is evident from the reported results that the indenter breaks the MT before the shell touching the other side. As such, our simulation results differ significantly from the reported results. It can be inferred that the indenter speed has strong effect on MT’s damage. Analyzing the total accumulated potential energy in the system, it can be inferred that the applied force mostly transformed into potential energy.

### Expansion Test

Since neither the stretching nor the bending test enables dimer separation in the lateral direction, it can be argued that only hydrostatic tension type loading can invoke such type of deformation mode. To simulate hydrostatic expansion, we inserted an artificial cylinder inside the MT and incrementally increased its radius. The cylinder is defined by the same surface potential as in the bending test. (See Method section for more detail). The incremental increase of radius size forces the expansion of the MT. We used four *αβ*-dimer ring sections in this expansion test. Only one simulation was performed. The radius of the cylinder started at 5 nm. It expends with a rate of 10 m/s and stops at 15 nm. The total simulated time was 1 ns.

**Figure 6:**
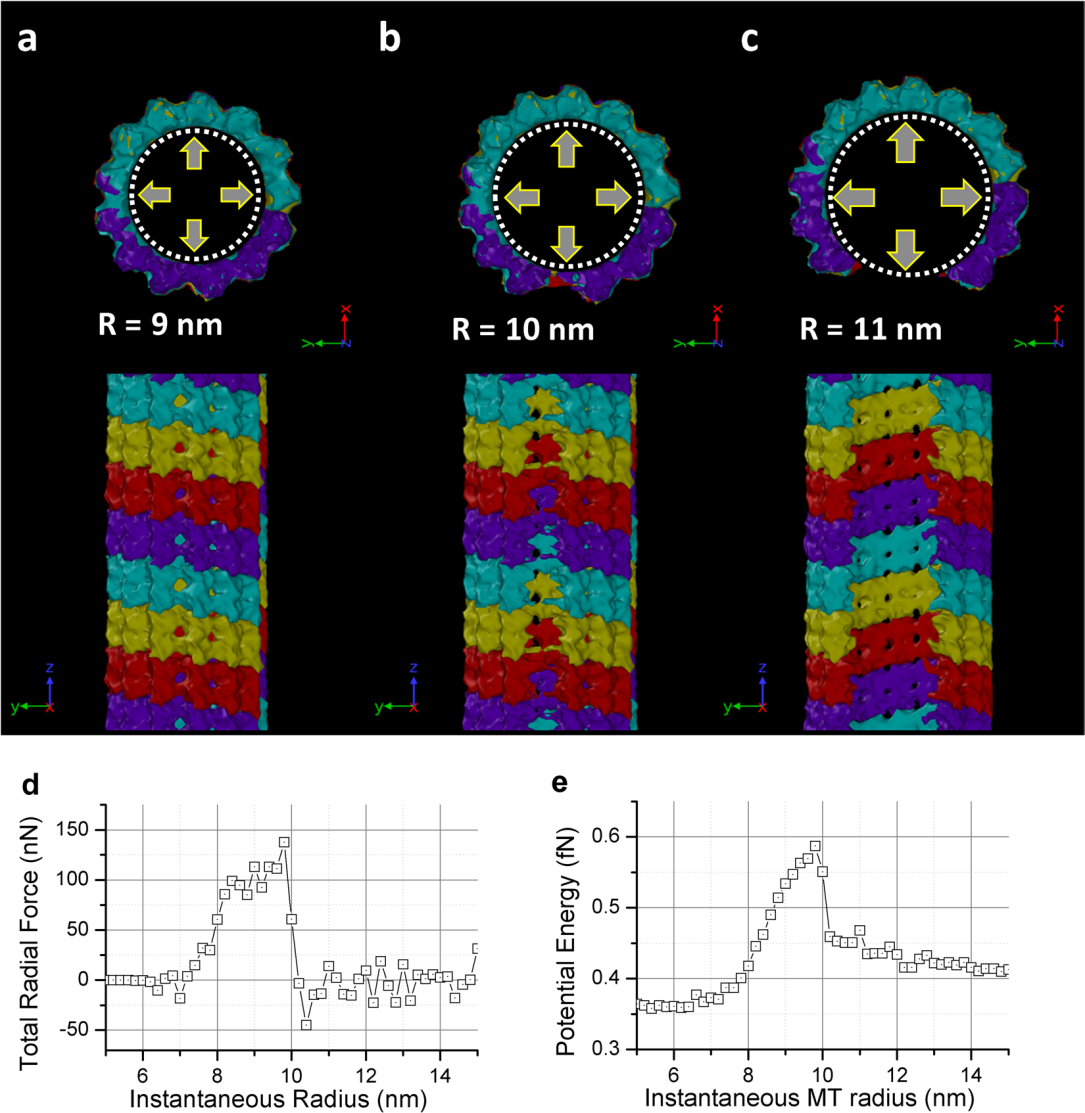
(**a**–**c**) The deformed MT after the expansion test. The dotted line indicates the artificial expander we inserted to do the test. (**d**) Force recorded on the expander during the expanding test (radio direction, sign reversed). (**e**) The total potential energy of the simulated system under the expanding test. For both figures, data points are logged with a 20 ps interval.

The results are shown in Fig. 6. It is evident that the hydrostatic failure of MT occurs near 90 degrees apart from the alpha-beta-dimer ring seaming junction, which indicate that the seaming junction is comparatively stronger than regular dimer to dimer tangential link.

For each increment in MT radius, we computed the total radial force on MT and the total potential energy. The results are shown in Fig. 6d,e. It shows that the energy required to break 4 MT dimer rings (~17 nm) is around 2.4 fJ. It is comparable to the stretching test. However, for the stretching test, the breakage is longer (~62 nm), which indicates the tangential link is roughly three to four times stronger.

## Conclusion

From this study, the following conclusions can be drawn:

- From the stretching and the expansion test, we found that the MT always breaks at the dimer interface. Failure is always preceded by interfacial crack(s) formation/delamination. It can be observed in the stretching test that crack only propagates along the dimer-dimer interface. Even when a crack meets protofilament-dimer ring junction, it deflects and continues to propagate along the nearest dimer-dimer interface.
- For bending under under high strain rate, MT bends and locally buckles (shell buckling) without showing any crack. The shell near the side of the buckling is bent nearly 180 degrees and touches each other on the inside.
- We found that the energy barrier required to break an MT axially (by stretching) is lower than to break tangentially (by expansion). It contradicts with many reported results where it is shown that the thermodynamic disassembly of MT occurs via protofilaments segregation. We believe, high-strain loading of MT does not provide sufficient time to the tangential junctions to release its energy as fast as the axial junctions.
- The axial strain-at-failure of MT for high strain rate loading is much smaller compared the strain-at-failure at lower strain rate (16% vs 50%). Moreover, the total force required to bend MT via high rate loading is roughly 10 time larger than to bend it via quasi-static loading. In short, MT is much stronger and more rigid at high strain rate.
- Comparing all simulation results, it can be argued that the more possible breaking mechanism of MT due to a nearby cavitation implosion is by stretching. The axial stress and axial strain at failure of MT is roughly 20–30% and 150 MPa, respectively.

It can be inferred from this study that cavitation implosion occuring somewhere adjacent to axon is capable of damaging and breaking axonal microtubule. Due to simulation restrictions, results reported in this paper can only predict and capture events that take place on the order of nanoseconds at the temporal level and nanometers at the spatial scale. The damage projected in this paper may propagate and fully disassemble the MT or repair itself through the thermodynamic assembly/disassembly process. Studies on this aspect are currently on going.

## Method

The MT structure used in this study is obtained from the literature^24^. It includes a helically arranged ring of 13 pairs of alpha and beta tubulins dimmers (along with 13 GTP, 13 GDP, and 13 magnesium ions at the interim junctions), which is a single periodic section of the MT. By copying these coordinates along the length direction (using periodic boundary conditions), one can form an infinitely long MT. We have equilibrated the periodic structure for another 1 ns to further minimize the potential energy. The equilibration was set with a targeted temperature of 310 K and 0 atm pressure at the z direction. Then we duplicate the section to build the MT with the desired length. Each periodic section contains a total of 174655 atoms.

The interatomic potential has been adopted from the CHARMM36 with CMAP^39,40^. The potential for GTP and GDP have been adopted from ATP and ADP as suggested in the literature^24^. The simulation is carried out by LAMMPS^41^ on STAMPEDE 2 super computer. The implicit water solvent model is adopted from LAMMPS (lj/charmm/coul/charmm/implicit). This type of model modifies the Coulombic energy by an extra *r*^−1^ power. For this implicit water model, no long range Coulombic force is required. All models are equilibrated for another 1 ns (LAMMPS NPT) with a target temperature of 310 K and 0 atm pressure at the z direction (except the bending case) after the duplication. The damping constant of thermal state during the equilibration is set to 50 fs and 1 ps for the barostat. A direct comparison between the explicit solvent and implicit solvent method is provided in the supplementary material.

During the stretching test, at each time step, we have scaled the size of the simulation sub-domain and the atomic positions proportionally to conform with to the desired strain rate. Since the strain rate simulated is much lower than the speed of atoms reaching the local minimum, no undesired artifact was observed.

For the bending test and the expansion test, artificial cylinders were placed into the model. The cylinder surfaces are assigned with 9–3 Lennard-Jones style potential with the depth of the potential well of 1.054 kcal/mol at a distance of 0.2574 nm. All three tests were set at constant temperature of 310 K with fixed size domain unless we artificially change it (LAMMPS “NVT” command). The damping constant of thermostat is set at 50 fs.

The surface plot in Figs 3, 5a–c and 6a–c is done by OVITO^42^. The concept of the method used in creating the surface is based on using a spherical probe to define the region that the probe can enter without touching the atoms. In our case, we chose the radius of the probe to be 0.3 nm.

### Data availability

The simulation datasets generated during and/or analyzed during the current study are available from the corresponding author on reasonable request.

## Electronic supplementary material

Stretching Test: Strain Rate = 0.025 × 109 1/s (View 1)

Stretching Test: Strain Rate = 0.025 × 109 1/s (View 2)

Stretching Test: Strain Rate = 1 × 109 1/s (View 1)

Stretching Test: Strain Rate = 1 × 109 1/s (View 2)

Stretching Test: Strain Rate = 1 × 109 1/s (View 3c)

Stretching Test: Strain Rate = 4 × 109 1/s (View 1)

Stretching Test: Strain Rate = 4 × 109 1/s (View 2)

Bending Test: Strain Rate = 1 × 109 1/s

Expansion Test: Strain Rate = 1 × 109 1/s

Supplementary Material
