# Supplementary material for: Damage and Failure of Axonal Microtubule under Extreme High Strain Rate: An In-Silico Molecular Dynamics Study
Source: Sci Rep. 2018 Aug 16;8:12260. doi: 10.1038/s41598-018-29804-w (PMC6095851; doi:10.1038/s41598-018-29804-w)
Supplement: Supplementary file 10 — Supplementary Material [file 41598_2018_29804_MOESM10_ESM.docx]

**Damage and Failure of Axonal Microtubule under Extreme High Strain Rate: An In-Silico Molecular Dynamics Study**

Yuan-Ting Wu, Ashfaq Adnan

Department of Mechanical and Aerospace Engineering, The University of Texas at Arlington, Arlington, TX 76019.

**Supplemental Material**

Whenever a MD simulation involves solvation, there are two ways simulation can be conducted, either by the implicit or the explicit solvent method. In the explicit solvation method, a simulation box contains explicitly defined water molecules in the system. On the other hand, in the implicit solvation technique, a simulation box does not contain any water molecules. The interaction between the implicit solvent and the simulated molecule is replaced by analytical descriptions. Many distinct models have been proposed, ranging from simple models that modify the long-range columbic force according to the solvent polarity, to complex models involving the mean force potential built by the solvent[^1^](#_ENREF_1)^,^ [^2^](#_ENREF_2). Since surface forces may affect overall mechanical behavior of a biological material, it is important to validate the applicability of the implicit solvent method with respect to the loading type, magnitude and rate imposed on the immersed molecule. While some comparative studies of explicit vs. implicit solvent simulations of various charged molecules are available in the literatures, a direct study on the applicability of implicit solvent method to simulate the mechanical response of microtubules/tubulins at different strain rate loadings is lacking. Such study is relevant here because, in the current study (main article), we performed all simulations with an implicit solvent model.

Structurally, a MT is a long, hollow, cylindrical tube composed of a staggered and parallel assembly of 13 protofilaments (PFs). Each PF is formed by end-to-end polymerization of *αβ* - tubulin heterodimers. It should be noted that in vitro polymerization of tubulin may yield MTs with a range of PF counts (between 8 and 17)[^3^](#_ENREF_3)^,^ [^4^](#_ENREF_4), but most microtubules have 13 or 14 PFs. As such, the model we considered in the main manuscript is representative of one of the many configurations seen in nature.

In this supplementary material, instead of modeling the entire MT, we have performed benchmark simulations with periodic 2-PF aggregate as a target molecule to examine whether the explicit/implicit solvent has any impact on its mechanical behavior at different strain rates. As outlined in reference[^5^](#_ENREF_5) , an infinitely long MT can be built by specifically assembling the “N-type” and “S-type” PF aggregates. It can be revealed from Fig. SI-1 that we have modeled the “N-type” PF aggregate and performed MD simulation with the implicit and explicit solvation techniques. Since in the reference[^5^](#_ENREF_5), all model were simulated with the explicit solvent method, some part of our simulation results can also be compared with that study.

We maintained identical box size, initial target molecule size and simulation conditions in both explicit and implicit solvent simulations. Periodic conditions are applied in all directions. To maintain consistent water density in the explicit solvent system, the lateral dimensions (along x-y plane) of the simulation box were constantly adjusted when the tensile loading was applied along the longitudinal direction (along z-direction). To keep overall charge-neutrality, appropriate number of potassium ions are added to the system. The simulation domain contained a total of 150,359 atoms. The standard CHARMM potential is used to define the interatomic interactions. The TIP3P model are used to define the water molecules. For the implicit solvation, the “LAMMPS CHARMM implicit” method is utilized. In this method, the range of coulombic interactions between the water and the target molecules are modified by adjusting the order from 1/r^2^ to 1/r. Such adjustment eliminates the requirement of Ewald Sum or the Potential of Mean Force type long-range electrostatic interactions. It should be noted that while such model makes larger and longer simulation more feasible, the method ignores the solvent-surface charge layer interactions. The model contained a total of 26,870 atoms. Both systems are first equilibrated for 1 ns at 310 K before any mechanical load is applied. We noticed that the final equilibrated length of PF-aggregate obtained by the two methods is not identical. In particular, the equilibrated length (7.812 nm) of the implicitly-solvated PF aggregate is roughly 7% shorter than the equilibrated length of the explicitly-solvated PF aggregate. This is somewhat expected because surface free energy is present in the implicitly-solvated system but absent in the explicitly-solvated system.

To estimate the surface free energies and the associated stress values, consider the PF aggregate is confined inside a rectangular box of height H, width 2D and thickness D, as shown in the Fig. SI-2. There are four tubulin monomers in each PF aggregate and each of the tubulin monomer could be assumed as a sphere of diameter D, as shown in Fig. SI-2(b). In the implicit solvent system, these spheres are exposed in vacuum, which, in turn, gives rise to surface tension force.

To obtain a simple relation between the surface tension and the resultant force, one of the spheres are cut open, as shown in Fig. SI-2(c). To maintain static equilibrium, the surface tension force must be balanced with the internal resultant force along the z direction (as plane normal to z is periodic). By dividing the resultants by the area on which they are acting, the internal stress components σ_z_ can be obtained.

Based on the free body diagram shown in Fig SI-3(c), the surface force F is related to the internal stress component σ_z_ as follows:

$F=2\pi r\gamma=-\sigma_{z}(\pi r^{2})$ (1)

Rearranging (1), the expressions for $\sigma_{z}$ can be obtained as,

$\sigma_{z}=-\frac{2\gamma}{r}$ (2)

The radius of tubulin is widely known and can be taken as *r* = 2.8 nm. Since the MT surface is weakly hydrophilic, we used the surface tension of water to estimate the excess surface tension on the implicit solvated MT surface, which is widely known as $\gamma=72 mN/m$ at around 310 K. Using these values, the internal stress component along the z direction can be computed as $\sigma_{z}=-51.4$ MPa.

The surface tension induced stress is very comparable to the compressive stress developed in the explicit system (~60 MPa vs ~52 MPa) when both systems have same box dimension. Since the surface tension induced stress is directly proportional to the radius of the sphere, it can be argued that the implicit vs explicit stress differences can be estimated by plotting stress difference as a function of r, as shown in Fig. SI-3. It can be observed that the effect of surface tension induced stress difference diminishes with larger length scales. We argue that the implicit vs explicit stress-strain relation should match very well when the surface induced stresses are accounted for in the comparison.

Since the strain rate in MD is typically calculated by dividing the initial length of the system by the applied velocity, to directly compare implicit vs explicit method at different strain rate, it is necessary that the initial length of the two systems be same. As such, we contracted the length of the explicitly solvated system by 7% before applying any mechanical loading. We acknowledge that such reduction in length generates residual stress in the system. We, however, have shown earlier that the generated residual stress is equivalent to the stress generated due to surface tension.

We tested three different stretching strain rates, namely 0.25 x 10^9^ s^-1^, 1 x 10^9^ s^-1^, and 4 x 10^9^ s^-1^. These strain rates are identical to what we applied on MT. In Fig. SI-1, it is shown that both the implicitly and explicitly solvated PF aggregates break between the dimers. It can be observed that the implicitly solvated aggregates stretched more at the αβ dimer interface when compared with the explicitly solvated aggregates.

In Fig. SI-4, the strain-rate dependent stress-strain responses along the z-direction are shown. The total virial stress on zz direction ($\sigma_{zz}$) is calculated using the effective cross section area of the PF aggregates (50 nm^2^). Since the initial length of the explicitly-solvated system is fixed at a length that is shorter than its relaxed stat, it is already under compression (roughly about 60 MPa). The stress-strain plots shown in Fig. S4 are developed by subtracting the initial stress (~ 0 MPa for implicit case but ~ - 60 MPa for explicit case) from the generated stress. Interestingly, it can be observed from Fig. S4 that the initial stress-strain response and the failure stresses are very comparable between the explicitly and implicitly solvated PFs. The post-failure stress-strain responses up to 20% strain are also in good agreement for the 0.25 x 10^9^ s^-1^ and 1 x 10^9^ s^-1^ loading cases. In the 4 x 10^9^ s^-1^ loading case, the post-failure stress-strain relation of the implicitly solvated system differs from the explicitly solvated system. Both systems exhibit strong strain rate dependency.

In summary, our benchmark study confirms following:

- There is slight difference in the equilibrated lengths and overall stress-strain responses of PF aggregates when simulated under implicitly-solvated and explicitly-solvated system.
- We argued that the difference in the stress-strain curve is due to surface tension effect that is only present in the implicit-solvent system. We have shown that by conducting simulations with identical system dimension, the implicit vs explicit stress-strain curves are very comparable.
- Readers should use caution when interpreting the stress-strain response obtained from implicit-solvent simulations. Without any surface-tension effect correction, the implicit system might overestimate the peak stress. The initial stress-strain response seems to be not affected by the surface tension effect.

Figure SI-1. **a**. Schematic of the benchmark simulation setup. The “N-type” – PF-aggregate^5^ modeled here can be considered as one of the primary building blocks of MT. The other building block is the “S-type” aggregate. **b**. MD snapshots taken from the stretching simulation performed at 1 x 10^9^ s^-1^ strain rate.


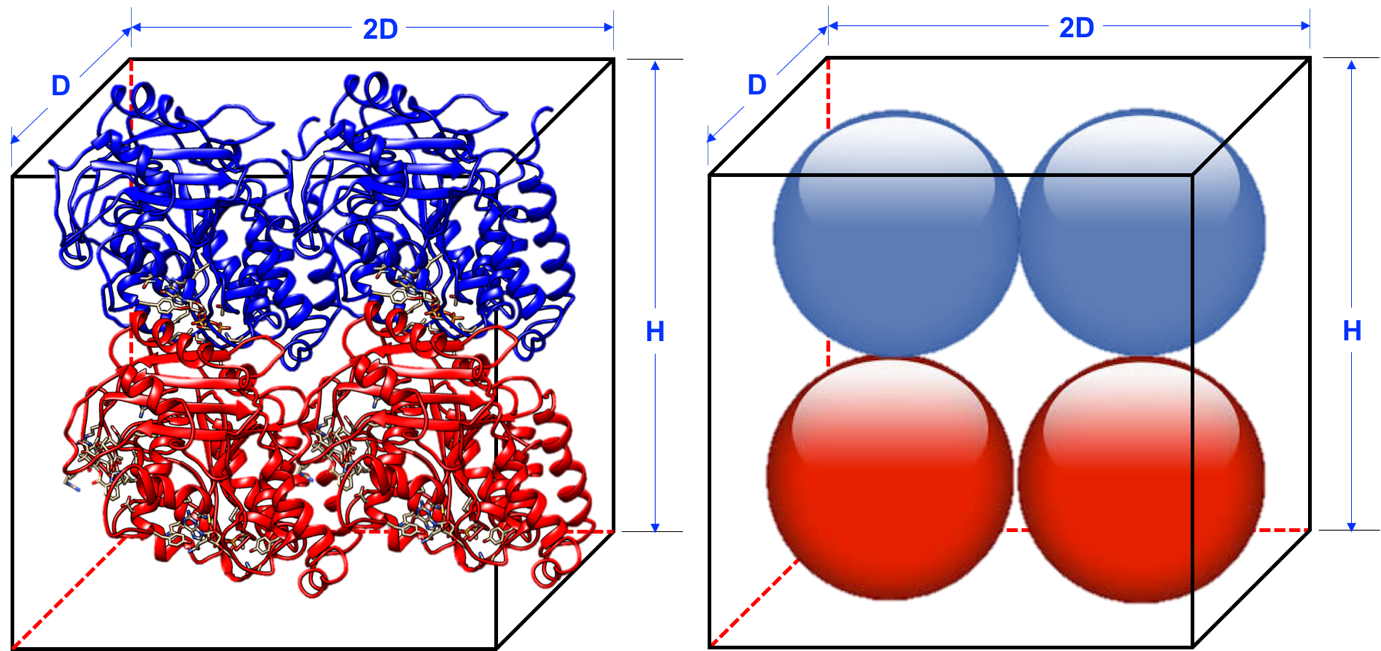


1. (b)

(c)

$$2\pi r\cdot\gamma$$

$$\sigma_{z}\pi r^{2}$$

**Surface tension force**

**Internal resultant resultantd**

Figure SI-2: (a) Simulation box confining the PF-aggregate. In the implicit solvent system, the outer surfaces of the tubulin monomers are exposed and give rise to surface tension. (b) each tubulin monomer is replaced by a sphere to simplify surface tension estimation. (c) One of the spheres are cut into half to show force balance between the surface tension force and the internal resultant.


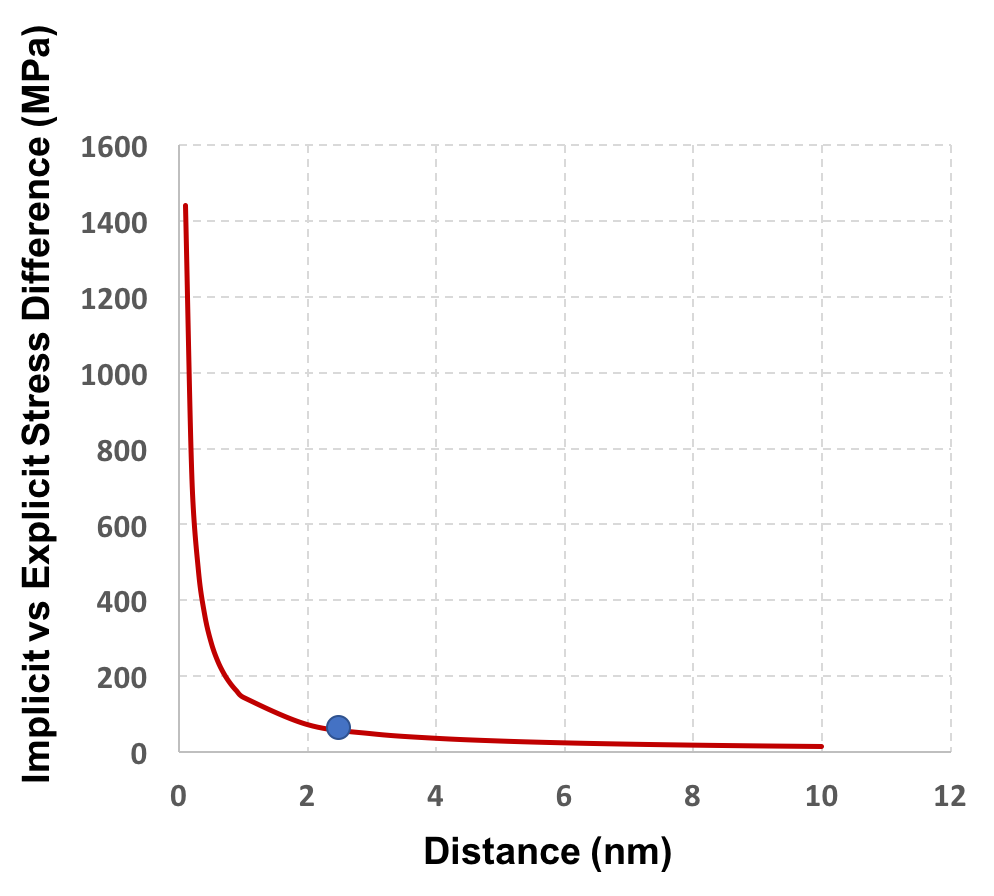


Figure SI-3: Differences in stress values when simulations are separately done with explicit and implicit system. Note that the stress difference arises solely because of the surface tension induced stress generation. The blue dot corresponds to PF aggregate length and the associated stress difference.

Figure SI-4. Pressure profile during the stretching benchmark. The cross section are of the two tubulin dimmer is approximated with 50 nm^2^

Reference:

1. Chen, J., Brooks III, C.L. & Khandogin, J. Recent advances in implicit solvent-based methods for biomolecular simulations. *Current opinion in structural biology* **18**, 140-148 (2008).

2. Kleinjung, J. & Fraternali, F. Design and application of implicit solvent models in biomolecular simulations. *Current opinion in structural biology* **25**, 126-134 (2014).

3. Chrétien, D., Metoz, F., Verde, F., Karsenti, E. & Wade, R. Lattice defects in microtubules: protofilament numbers vary within individual microtubules. *The Journal of cell biology* **117**, 1031-1040 (1992).

4. Lodish, H. et al. Molecular cell biology 4th edition. *National Center for Biotechnology Information, Bookshelf* (2000).

5. Wells, D.B. & Aksimentiev, A. Mechanical properties of a complete microtubule revealed through molecular dynamics simulation. *Biophysical journal* **99**, 629-637 (2010).

6. Park, H.S. & Klein, P.A. Surface Cauchy-Born analysis of surface stress effects on metallic nanowires. *Physical Review B* **75**, 085408 (2007).

7. Gibbs, J.W. ART. LII.--On the Equilibrium of Heterogeneous Substances. *American Journal of Science and Arts (1820-1879)* **16**, 441 (1878).

8. Cammarata, R.C. Surface and interface stress effects in thin films. *Progress in surface science* **46**, 1-38 (1994).
